# Upregulation of GLRs expression by light in Arabidopsis leaves

**DOI:** 10.1186/s12870-022-03535-7

**Published:** 2022-04-15

**Authors:** Anna Hebda, Aleksandra Liszka, Aleksandra Lewandowska, Jan J. Lyczakowski, Halina Gabryś, Weronika Krzeszowiec

**Affiliations:** Department of Plant Biotechnology, Faculty of Biochemistry, Biophysics and Biotechnology, Jagiellonian University in Kraków, Gronostajowa 7, 30-387 Kraków, Poland

**Keywords:** Blue light, Cryptochrome, GLR, Phototropins, Phytochromes, Red/far red light

## Abstract

**Background:**

Glutamate receptor-like (GLR) channels are plant homologs of iGluRs, animal ionotropic glutamate receptors which participate in neurotransmission. GLRs mediate plant adaptive processes and photomorphogenesis. Despite their contribution to light-dependent processes, signaling mechanisms that modulate *GLR* response to light remain unknown. Here we show that leaf expression of 7 out of 20 Arabidopsis *GLRs* is significantly up-regulated by monochromatic irradiation.

**Results:**

Our data indicates that both red and blue light stimulate the expression of selected *AtGLRs*. Using a photosynthesis inhibitor and different irradiation regimes, we demonstrated that retrograde signaling from photosystem II is unlikely to be involved in light-dependent *GLR* up-regulation. Analysis of transcriptional patterns in mutants of key photoreceptors allowed us to observe that both phytochromes and cryptochromes are likely to be involved in the control of light-dependent up-regulation of *AtGLR* expression, with phytochromes playing a clearly dominating role in this process.

**Conclusions:**

In mature Arabidopsis leaves, phytochromes, assisted by cryptochromes, mediate light-driven transcriptional up-regulation of several genes encoding GLR proteins. Since GLRs are known to be involved in a wide range of plant developmental processes our results provide mechanistic insight into how light may influence plant growth and development.

**Supplementary Information:**

The online version contains supplementary material available at 10.1186/s12870-022-03535-7.

## Background

Plant membranes contain numerous nonselective ion channels. Among them, GLutamate Receptor-like (GLR) channels are widely studied in recent years due to their proposed role in a variety of physiological processes ranging from control of seedling growth [1] to the response of mature plants to pathogen infection [2]. GLRs are multimeric, ionotropic, ligand-gated receptors [1]. *Arabidopsis thaliana* genome contains 20 genes encoding GLR channel components [3] which, depending on the choice of sequences used for the phylogenetic analysis, are divided into three [4] or four [5] clades. Plant GLRs are homologous to the mammalian iGluRs (ionotropic glutamate receptors), which, in animals, play an important role in cognitive processes such as memory and/or learning [6]. This homology facilitates plant GLR study thanks to the availability of a range of chemical agonists and antagonists for animal iGluRs. A growing number of experimental data shows the involvement of GLRs in plant physiology [7] but the control of GLR contribution to physiological processes remains unexplored.

GLR channels may be involved in light signal transduction. Two light-regulated processes were evaluated in the very first publication about GLR channels in plants: hypocotyl elongation and de-etiolation of *A. thaliana* seedlings [1]. In these experiments, a specific iGluR inhibitor: DNQX, reduced blue-light induced shortening of etiolated hypocotyls. As DNQX did not affect seedling growth in darkness, it must have impaired a light-dependent process. Moreover, the application of the inhibitor reduced the chlorophyll content in the treated seedlings. Similarly to the application of DNQX inhibitor, treatment with BMAA, an agonist of iGluRs, reduced light-induced shortening of hypocotyls; BMAA also decreased the opening of cotyledon arcs in light [8]. Unlike wild type (WT) plants, hypocotyls of Arabidopsis photomorphogenic *det3* mutant responded to neither DNQX nor BMAA [9], which suggests a link between DET3, a component of a developmental light switch in plants, and GLRs in the control of seedling growth. Expression of *GLR* genes in flowers [4, 10] suggests that the channels may play a role in flowering, a canonical photomorphogenic process [11]. In addition to that, specific GLRs were linked to processes which involve light signaling. For example, *At*GLR3.1 is involved in stomatal movements [12], a process regulated by phytochromes A and B, cryptochromes, and phototropins [13]. *At*GLR1.1 is a C/N ratio regulator [14]. Noteworthy, high nitrogen levels delay flowering via light-dependent ferredoxin-NADP+-oxidoreductase (FNR1) and CRY1 [15]. *At*GLR3.5 was shown to be involved in leaf senescence [16] that was demonstrated to require phytochrome-interacting transcription factors, PIF4 and PIF5 [17].

Despite evidence supporting light dependence of *GLR* activity, it is unknown how this light control is exerted. The first stage of this control may occur at the level of gene expression. One of the two most frequently occurring *cis*-acting elements in *AtGLR* promoter regions is GATA, a motif linked with light-dependent gene regulation in plants [18]. GATA motifs have been found in promoters of several photosynthesis-related genes, such as *CAB (chlorophyll a/b-binding protein)*, *GAP (glyceraldehyde-3-phosphate dehydrogenase)*, *RBCS* (*Rubisco small subunit*) [19]. Only two promoters (those of *AtGLR2.4* and *AtGLR2.1*) do not contain GATA motifs. Instead, they contain other light-regulated sequences: an Ibox and BoxII [18]. Therefore, based on genomic sequence alone, it is likely that light signals influence *GLR* expression in plants.

To take the first step towards understanding how light influences the activity of selected *GLRs* in Arabidopsis leaves, we conducted a comprehensive expression profiling analysis using real-time PCR. We compared *AtGLR* expression profiles in WT and mutants of six photoreceptors responsible for photomorphogenic development: four single, *phyA*, *phyB*, *cry1,* and *cry2* and two double, *cry1cry2* and *phot1phot2* mutants. Irradiation with red light (RL) and blue light (BL) and a photosynthesis inhibitor were used to understand the signaling pathway by which light controls *AtGLR* expression. Our work indicates that mRNA levels of most *AtGLRs* in leaves increase upon irradiation and that this increase is likely to be controlled by photoreceptors, chiefly by phytochromes, rather than by retrograde signaling from the photosynthetic apparatus. One of the greatest strengths of our study stems from the use of whole mature plants and their irradiation with defined monochromatic light regimes. This enabled us to look into the mechanism of light regulation of *GLR* transcript levels at the major stage of plant ontogenesis.

## Results

To get an insight into the mechanism through which light may regulate *AtGLR* expression, we analyzed the effects of two canonical spectral regions, red and blue, on leaf transcriptional profiles. Red and blue parts of the visible spectrum activate both photosynthesis and photomorphogenesis, the latter mainly controlled via two key photoreceptor families: phytochromes (R-FR) and cryptochromes (BL). Twelve Arabidopsis *GLRs* that showed detectable expression in leaves were chosen for further studies.

### Red and blue light expression pattern of *AtGLRs* in wild type plants

**Fig. 1:**
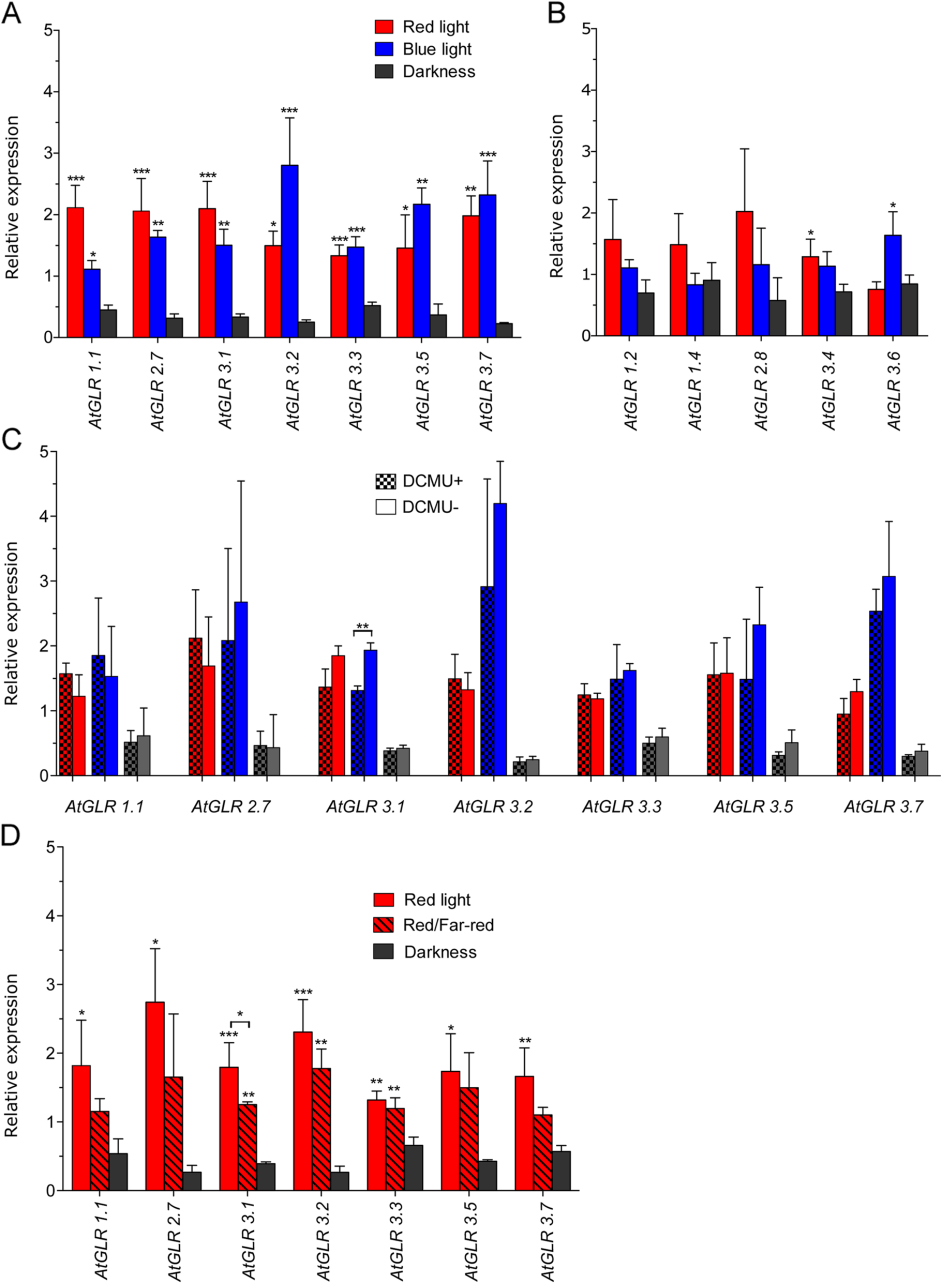
The effect of light on the relative expression of *GLRs* in mature leaves of wild type Arabidopsis. Four-week-old soil-grown plants were dark-adapted for 16 h and illuminated according to different light regimes. (**A**, **B**) plants were illuminated for 3 h with equimolar red light or blue light or left in darkness. Light-driven transcriptional up-regulation of *GLR* genes was more than 3-fold (**A**) and less than 2-fold (**B**) as compared to darkness. (**C**) plants were treated with 50 μM DCMU or left without treatment (control) and illuminated with red light or blue light as in (**A**, **B**). (**D**) plants were illuminated for 3 h with continuous red or 3 h irradiation with R/FR alternated every 2 min. The results collected in graphs represent means of three biological replicates with error bars denoting standard deviation (SD). Each replicate contained leaves from two plants. Asterisks indicate significant differences (* *p* ≤ 0.05, ** *p* ≤ 0.01 and *** of *p* ≤ 0.001) determined by One way ANOVA with Dunnett’s test (*n* = 3) in A, B and with Tukey’s test in D. The differences between means were calculated with a two-tailed unpaired Student’s t-test in C

Arabidopsis WT plants (ecotype Columbia) were exposed to RL or BL and *AtGLR* transcription levels were determined in mature leaves. Transcript levels of *AtGLR1.1*, *AtGLR2.7*, *AtGLR3.1*, *AtGLR3.2*, *AtGLR3.3*, *AtGLR3.5*, *AtGLR3.7* genes in light-treated leaves were more than 3 times higher than in leaves kept in the dark (Fig. 1A) and all differences were statistically significant. Thus, further analysis focused on these genes. The remaining five leaf-expressed *AtGLRs* (Fig. 1B) were only weakly upregulated by light, with induction not exceeding 2-fold or not being statistically significant.

*AtGLR1.1*, *AtGLR2.7, AtGLR3.1* were strongly upregulated by RL showing 5-, 7-, and 6-fold increases, respectively. The expression of *AtGLR3.2, AtGLR3.5, AtGLR3.7* increased over 10-, 6- and 10-fold, respectively, after BL treatment. For *AtGLR3.3* both light ranges generated a similar transcriptional up-regulation. In summary, our results show that blue and red monochromatic light treatments increase leaf expression levels of several *AtGLRs.*

### *GLR* expression in leaves with inhibited light phase of photosynthesis

To establish if photosynthesis contributes to the observed light-driven up-regulation of *AtGLR* expression, we analyzed the transcript levels following DCMU treatment of leaves in WT (Col.) Arabidopsis. DCMU is a specific, long-acting photosystem II inhibitor which blocks the linear electron flow in photosynthesis [20, 21]. Fifty μM DCMU was previously shown to halt photosynthetic oxygen evolution in Arabidopsis [22]. Thus, DCMU should reduce light-driven *AtGLR* up-regulation, if it is a photosynthesis-dependent process. The impact of DCMU on *AtGLR* expression was tested in RL, BL, and darkness. No difference was observed between DCMU treated and control samples for most *AtGLRs* (Fig. 1C). For *AtGLR3.1,* we observed a reduced extent of light-driven transcriptional up-regulation upon DCMU application. However, in both DCMU treated and untreated leaves light strongly stimulated *AtGLR3.1* expression when compared to that seen in leaves maintained in the dark. Importantly, in this experiment, we again observed that the levels of *GLR* transcripts in the dark are several times lower than in light. This validates our previous observations (Fig. 1A), that the expression of selected *AtGLR*s in leaves is stimulated by light.

### *GLR* expression upon red/far red light treatment

Phytochromes are prime RL plant photoreceptors. Since RL irradiation activates all members of the phytochrome family (phyA, phyB to phyE) and FR reverses only responses of phyB clade members, we used these two light ranges to investigate which phytochromes play a role in the control of *AtGLR* expression. Following standard procedures, short alternating pulses of RL and FR, with FR fluence rate exceeding that of RL by approximately two-fold, were used to test if phyB contributes to the observed transcriptional up-regulation [23].

Strong RL significantly up-regulated *AtGLR* transcript levels compared to these measured in leaves kept in the dark. Interestingly, a similar extent of up-regulation was obtained with nine times weaker RL, used in the current experiment, compared to that applied in the previous analysis (Fig. 1D). A significant decrease of expression level by FR was observed only for the *AtGLR3.1* gene, suggesting its control mainly by *phyB*. Although some reduction of expression by FR was also observed for *AtGLR1.1*, *AtGLR2.7* and *AtGLR3.7*, it was not statistically significant, which suggests the contribution of both *phyA* and *phyB* to the control of their expression.

### Red and blue light expression patterns of *AtGLRs* in photoreceptor mutants

To establish the contribution of particular photoreceptors to light-dependent transcriptional up-regulation of *AtGLRs*, we extended our investigation to four single mutant Arabidopsis lines, lacking functional copies of genes encoding phytochromes *phyA* or *phyB* or cryptochromes *cry1* or *cry2* and to double mutants *cry1cry2* and *phot1phot2. Landsberg erecta* ecotype was used as a control for cryptochrome mutants.

**Fig. 2:**
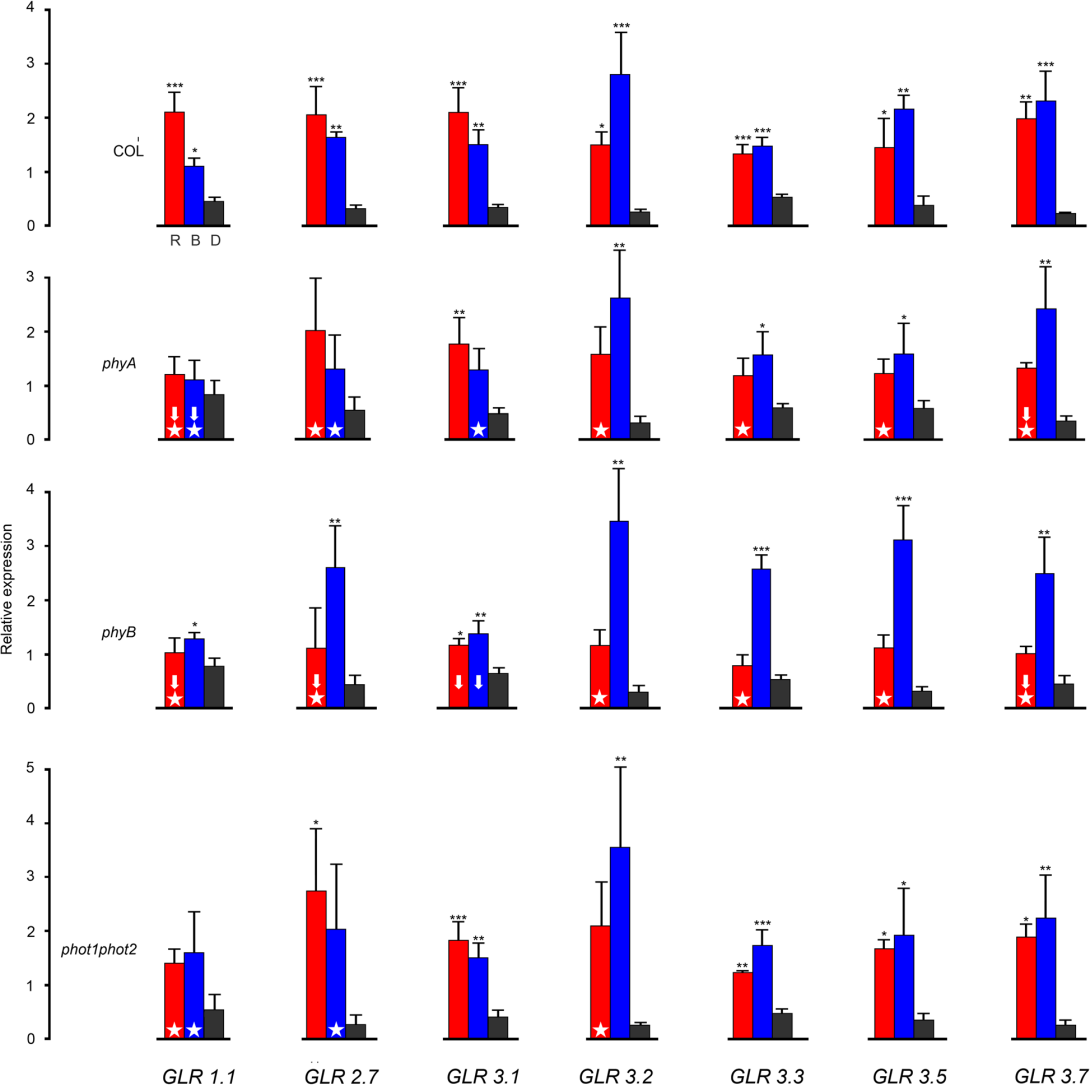
The effect of light on the relative expression of *AtGLRs* in mature leaves of photoreceptor mutant plants: *phyA*, *phyB*, *phot1phot2* compared with WT Arabidopsis (Columbia background, repeated from Fig. 1). Four-week-old soil-grown plants were dark-adapted for 16 h and illuminated according to different light regimes. Plants were illuminated for 3 h with equimolar RL or BL or left in darkness. The results collected in graphs represent means of three biological replicates with error bars denoting standard deviation (SD). Each replicate contained leaves from two plants. Asterisks indicate significant differences (* *p* ≤ 0.05, ** *p* ≤ 0.01, and *** of *p* ≤ 0.001) determined by One way ANOVA with Dunnett’s test (*n* = 3). Major differences between light-induced expression in WT and photoreceptor mutants are marked with white arrows. White stars emphasize the disappearance of statistical significance of the light effect in mutants. A detailed explanation is provided in the text

**Fig. 3:**
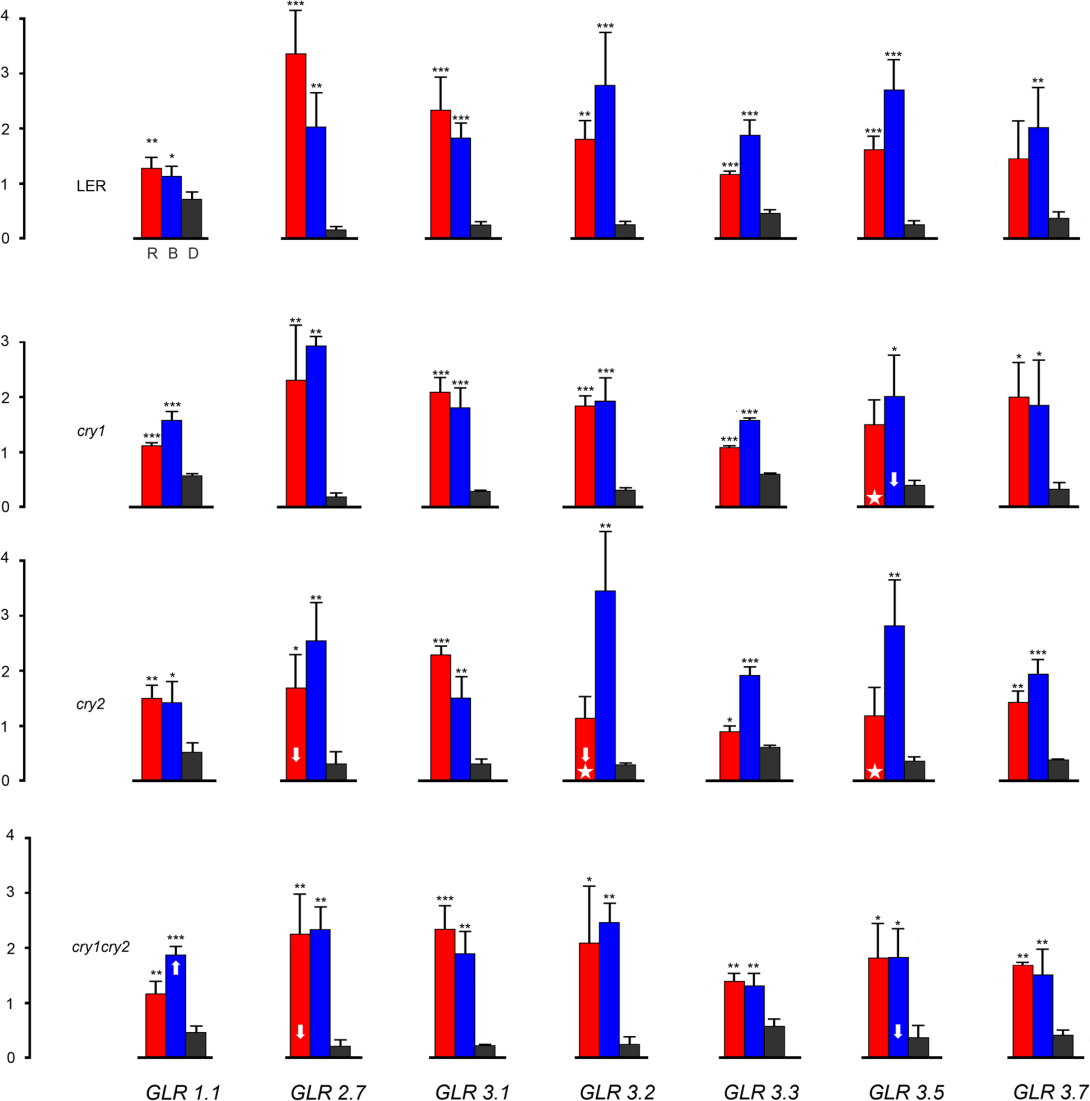
The effect of light on the relative expression of *AtGLRs* in mature leaves of photoreceptor mutant plants: *cry1*, *cry2,* and *cry1cry2* compared with WT Arabidopsis (*Landsberg erecta* background). Four-week-old soil-grown plants were dark-adapted for 16 h and illuminated according to different light regimes. Plants were illuminated for 3 h with equimolar RL or BL or left in darkness. The results collected in graphs represent means of three biological replicates with error bars denoting standard deviation (SD). Each replicate contained leaves from two plants. Asterisks indicate significant differences (* *p* ≤ 0.05, ** *p* ≤ 0.01, and *** of *p* ≤ 0.001) determined by One way ANOVA with Dunnett’s test (*n* = 3). Major differences between light-induced expression in WT and photoreceptor mutants are marked with white arrows. White stars emphasize the disappearance of statistical significance of the light effect in mutants. A detailed explanation is provided in the text

Effects of BL and RL on the relative expression of *GLRs* in mature leaves of mutants are summarized in Figs. 2 and 3. We compared changes of the relative expression (RL to D, BL to D) observed in the mutants in relation to the respective changes in WT plants. As the obtained expression patterns turned out to be complex we adopted two criteria to evaluate the effects of light and photoreceptor deficiency on the transcript level. Firstly, we calculated induction factors for transcripts of specific *AtGLR*s at given wavelengths (Additional file 2). The factors were calculated by dividing RL or BL expression by that seen in the dark. Induction factors for photoreceptor mutants were compared to those calculated for WT plants, and differences equal or greater than 2-fold between them are marked in figures with white arrows. Secondly, we examined the statistical significance of differences between light and dark expression levels for WT and mutant plants. White stars in Figs. 2 and 3 mark the disappearance of the statistical light effect in mutants.

As shown in Fig. 2, both criteria are concomitantly fulfilled in 3 cases: for *GLR1.1* and *GLR3.7* (for both phytochrome mutants) and *GLR2.7* (*phyB* only). Thus, phytochromes A and B appear to be crucial in the regulation of *GLR1.1 and GLR3.7* while the control of *GLR2.7* expression is dominated by phytochrome B. The dominating influence of phytochrome B is also visible for *GLR3.3* and *GLR3.1*, each of them fulfilling only one criterion. However, *GLR3.3* seems to be controlled also by phytochrome A, and *GLR3.1* only by phytochrome B (cf. expression upon red/far red light treatment).

The involvement of cryptochromes in the control of *AtGLR* expression (Fig. 3) is less evident than that of phytochromes. Since cryptochrome mutants used in this study are in the Ler ecotype we needed to firstly evaluate light-driven up-regulation of *AtGLR* expression in WT Ler plants. Noteworthy, RL and BL up-regulation of *AtGLR* expression in WT Ler, although being qualitatively analogous, shows quantitative differences when compared with WT Col-0 (cf. top rows in Figs. 2 and 3). In particular, in WT Ler plants, *GLR1.1* is only weakly up-regulated by light while the up-regulation of *GLR2.7* is much stronger than in WT Col-0. A distinct reduction of BL effect on expression, expected for cryptochrome mutants, is visible only for *GLR3.5*, in *cry1* and *cry1cry2*. For the remaining *AtGLRs,* the BL-induced transcriptional upregulation remains high despite cryptochrome deficiency in the studied mutants. Interestingly, for several *AtGLRs* the RL induced transcriptional upregulation drops markedly in cryptochrome mutants. This is visible for *GLR2.7* in *cry2*, *cry1cry2,* and, to a lesser extent, in *cry1,* as well as for *GLR3.2* in *cry2*. Moreover, for *GLR3.3* RL-driven up-regulation disappears in the *cry2* mutant.

It should be noted that a high level of BL up-regulation is maintained in cryptochrome mutants. This might suggest an involvement of other blue-light photoreceptors in the control of *AtGLR* expression. Phototropins are, apart from cryptochromes, the second major photoreceptor family which mediates BL effects in plants. Therefore, we decided to check the level of *AtGLR* expression in the phototropin mutant, *phot1phot2* (Columbia background), which lacks active copies of genes encoding both phototropins. The results are shown in Fig. 2, bottom row. Except for *AtGLR1.1*, the patterns of expression in *phot1phot2* do not differ from these observed for WT Col. This suggests that the contribution of phototropins to BL driven up-regulation of *AtGLR* expression is likely to be only marginal.

## Discussion

No consistent picture of *GLR* regulation and function is available, despite much original data and reviews being published on this gene family in recent years (see references in Table 1). The expression of all *GLR* genes was proposed to be light-dependent. However, even the basic data on organ expression profiles of *AtGLRs*, assembled in Table 1, differ between publications. In particular, different groups show diverse *AtGLR* expression patterns in leaves. Thus, it is necessary to determine *GLR* expression using a well-defined experimental system. As such, our analysis which consistently uses mature leaves and controlled monochromatic light conditions might be of interest to scientists investigating leaf-specific *GLR* channels and their physiological role.

**Table 1:**
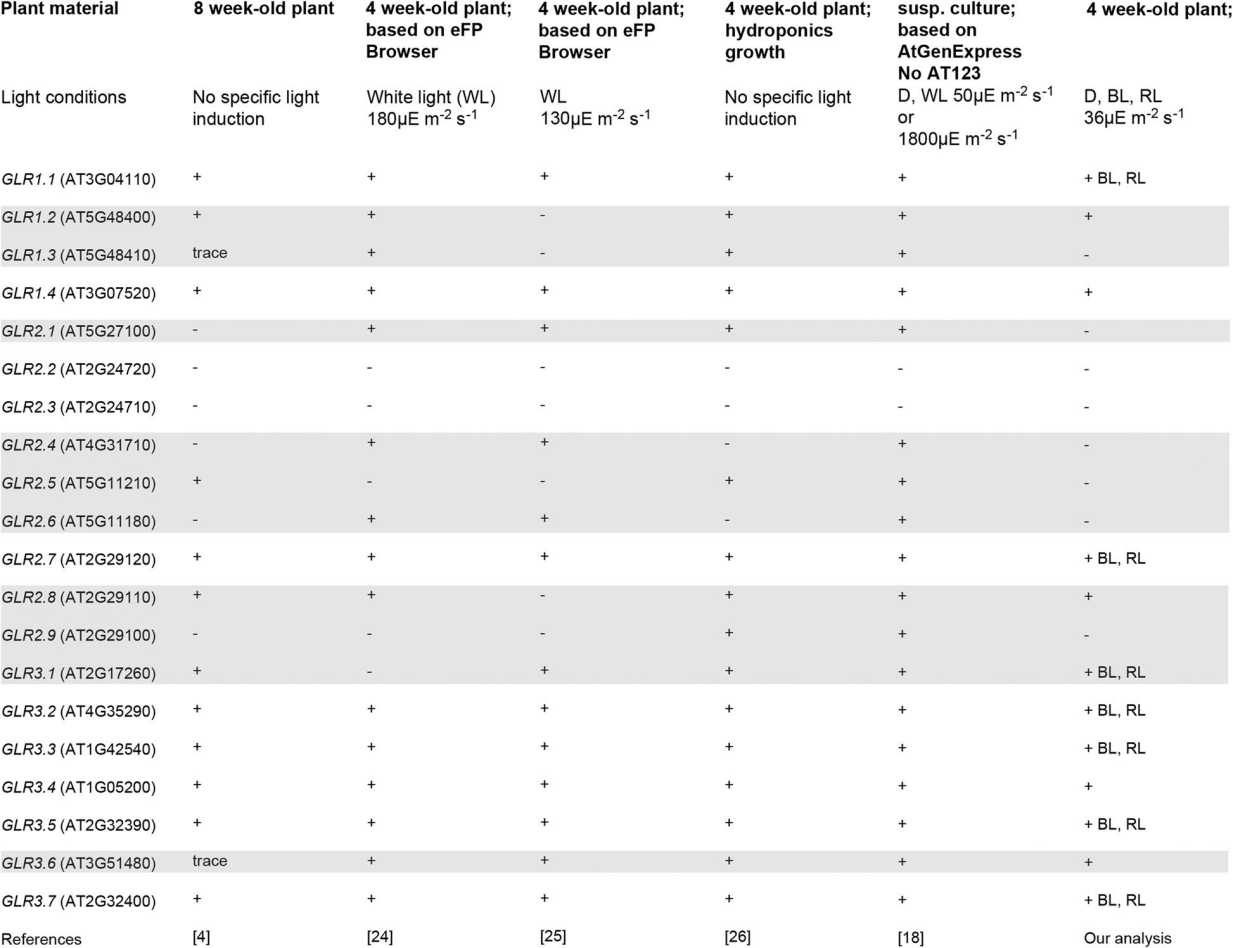
*AtGLR*s expressed in leaves - comparison of our results with those obtained by other groups. Expression in leaves is indicated by +, irrespective of its strength. Presence of any differences in the *AtGLR*s expression pattern is indicated with gray coloration of specific rows. Noteworthy, our results show that *AtGLR* representatives from all three clades are expressed in mature leaves

Our data on light-induced *AtGLR* up-regulation may seem contradictory to the results from previous microarray analyses, which showed different expression patterns for *AtGLR* in response to light ([18, 24, 25] cf. Table 1). For example, Roy and Mukherjee [18] reported that the expression of *AtGLR1.1, AtGLR2.7, AtGLR3.2,* and *AtGLR3.3* was greatest in the dark compared to that induced by low (50μE m^− 2^ s^− 1^) or high (1800μE m^− 2^ s^− 1^) light. In our case, mRNA levels for these genes were upregulated by light. It should be noted, however, that our experimental system is different from that used by Roy and Mukherjee [18]. Most importantly, the microarray analysis was performed on Arabidopsis cell suspension cultures, while we used whole plants and analyzed *GLR* expression in leaves. Gene expression in single cultured cells is specific and does not reflect processes occurring in cells embedded in tissues that are regulated at a whole plant level. Therefore, the results of the previous analysis are not fully comparable to our outputs.

Transcriptional control by light may be mediated by photoreceptors or by photosynthetic signaling. Irradiation with different light regimes, use of photoreceptor mutants, or application of DCMU photosynthesis inhibitor can all be used to elucidate which mechanism controls light-driven gene up-regulation [27]. The expression profiling in leaves treated with DCMU did not show substantial DCMU-dependent inhibition of light-driven transcriptional up-regulation. DCMU inhibits electron transport between PSII and PSI, impacting the acceptor side of PSII [20]. Since photosynthesis-derived retrograde signals from chloroplasts to the nucleus are unlikely to play a significant role in the regulation of *AtGLR* expression, we explored the contribution of photoreceptors to the control of this transcriptional process.

Our work is the first to analyze the role of photoreceptors in the light-dependent regulation of *GLR* expression. We detected RL-induced and BL-induced enhancement of *GLR* expression in leaves, which suggests the involvement of phytochromes and cryptochromes, respectively. This observation was followed by further experiments on mutants of these RL and BL photoreceptors. Transcriptional profiles of selected *AtGLRs* in these mutants suggest that their expression is controlled, in most cases, by more than one photoreceptor. None of the expression patterns obtained for different *AtGLRs* in WT and photoreceptor mutants matches another one, which suggests distinct molecular mechanisms for light stimulation of their expression.

Despite the complexity of the processes controlling *AtGLR* expression in light, our analysis using photoreceptor mutants may be summarized in the following way:

1. Reduced levels of RL up-regulation of *AtGLR* expression in phytochrome mutants imply a direct contribution of both phyA and phyB. This phyA and phyB co-regulation likely happens for all tested genes, except for *AtGLR3.1,* for which clear domination of phytochrome B is apparent as inferred from the strongly reduced RL stimulation only in the *phyB* mutant. In line with that, *AtGLR3.1* is also the only gene with statistically significant R-FR reversibility of expression in WT (cf. Fig. 1D).
2. The BL-induced transcriptional up-regulation is markedly reduced only for *GLR3.5* in *cry1* and *cry1cry2* mutants. For other genes tested, the reduction of BL-induced transcriptional up-regulation in cryptochrome mutants is much smaller or negligible. Moreover, in a *cry2* mutant, the up-regulation of *GLR2.7* and *GLR3.2* expression is significantly reduced in RL while it stays at the levels of WT in BL. Thus, no simple interpretation concerning the role of cryptochromes in boosting *GLR* expression is possible. No reduction of BL-induced transcriptional up-regulation of *AtGLR* expression in *cry1* and *cry2* mutants, as compared to WT, may be due to the redundancy of cryptochromes, a trait typical for these photoreceptors [28]. Closer examination of specific GLRs reveals that cryptochromes co-regulate their expression along with phytochromes. For example, the expression of *AtGLR1.1* is undeniably under the strong control of both phytochromes. Yet in the absence of cry1 and cry2 (i.e. in the double *cry1cry2* mutant), the BL enhancement of *AtGLR1.1* expression is even stronger than in the WT. This implies that cryptochromes act redundantly with each other, and antagonistically to phytochromes. Another mode of photoreceptor cooperation is apparent in regulating *AtGLR3.5* expression. Here, cry1 appears to cooperate with phyA, as reflected by the drop of BL- and RL-driven up-regulation in both cry1 and phyA single mutants. Similarly, although phyB is the main photoreceptor to convey the signal to *AtGLR2.7* up-regulation, phyA and cryptochrome(s) also appear to participate in this process. This may be inferred from the reduction of the BL transcriptional effect in *phyA* mutant as compared with WT and from a strong drop in RL-driven *AtGLR2.7* transcriptional enhancement observed in *cry2*.
3. Since *AtGLR* expression profiles in the *phot1phot2* mutant are similar to those seen in WT Col, phototropins are unlikely to be involved in light-driven transcriptional up-regulation of the studied genes.

Our results point to the cooperation of multiple photoreceptors in the control of light-induced transcriptional up-regulation of *AtGLR* expression. Similar phytochrome and cryptochrome cooperation was previously reported for the regulation of expression of genes encoding four transcription factors: HY5, HYH, SPA1, and SPA4 [29]. Having in mind that GLRs function as multimeric channels, the exact mechanism and biological significance of the observed light-controlled-expression may become apparent only when we establish the exact composition of functional GLR channels in plant cells. Another question that remains open is whether the final amount of GLR proteins is controlled only at the level of gene expression or if post-transcriptional control is also involved. This has been studied only for *AtGLR3.2* [10], where transcriptional control appears to be decisive for final protein levels.

## Conclusion

In mature Arabidopsis leaves, red and blue light up-regulate the transcription of several genes encoding GLR proteins. We demonstrate that this light-dependent up-regulation of GLR expression is mediated by phytochromes and cryptochromes, with the former ones playing a dominant role in the process. As such, our findings describe a direct link between light, the key environmental cue that regulates plant growth, and a family of genes known to be involved in diverse plant developmental processes.

## Methods

*Arabidopsis thaliana* WT (wild type) and mutant plants were grown in peat pellets (Jiffy International AS) in a growth chamber (Sanyo MLR-350H) at 23 ± 2 °C, 80% relative humidity, with a 10 h light /14 h darkness photoperiod, illuminated with fluorescent lamps (Philips Master TL-D-36 W/840, Osram L36 W/77 Fluora, Activa 172-36 W, Sylvania Gro-Lux F36W/GRO-T8), at an average fluence rate of 100 ± 20 μmol m^− 2^ s^− 1^. In all experiments, 4-week old plants were used. As the available cryptochrome mutants had been obtained in *Landsberg erecta* background, the results for *cry1, cry 2,* and *cry1cry2* were compared to that line. Seeds of *Arabidopsis thaliana* wild type Col-0 (ID: N60000) and *Landsberg erecta* (ID: NW20) were purchased from The Nottingham Arabidopsis Stock Centre (NASC). For experiments on light expression patterns of *AtGLRs* in photoreceptor mutants we used: *phyA-211* [30], *phyB9* [31], *cry1*, *cry2* and *cry1cry2* [32], *phot1phot2* - mutant was obtained by crossing *phot1* (SALK_088841C, NASC) with *phot2* [33].

### Red and blue light treatments

Plants, dark-adapted for 16 h (from 6 PM to 10 AM), were irradiated for 3 h with 36 μmol m^− 2^ s^− 1^ RL or BL. BL was obtained from LXHL-PR09 LEDs (Ledium Ltd. Hungary) with a maximum emission at 455 ± 20 nm (half-band width). RL was obtained from Luxeon Rebel ES LEDs (Philips Lumileds Lighting Comp.) with a maximum emission at 655 ± 14 nm. During the irradiation treatments, control plants were kept in darkness for the same time. Following light treatments, two leaves from two different plants were harvested and immediately frozen in liquid nitrogen. These two leaves were used for RNA extraction. All irradiations started at 10 AM and finished at 1 PM ± 15 min.

### Red/far-red light treatments

The involvement of phytochrome B in the regulation of gene expression was tested using far-red light (FR) in WT Arabidopsis plants. Following dark adaptation (16 h) alternated R/FR was applied for 3 h. 2 min RL (655 ± 14 nm) LED light of 4.6 μmol m^− 2^ s^− 1^ was followed by 2 min FR (730 nm ± 15 nm) LED light of 9.9 μmol m^− 2^ s^− 1^. Because of technical problems with obtaining higher intensities of FR, we used RL of lower intensity than in other experiments. All irradiations started at 10 AM and finished at 1 PM ± 15 min.

### DCMU treatment

*In planta* rosette leaves of Arabidopsis WT were dipped three times in 50 μM DCMU (3-(3,4-dichlorophenyl)-1,1-dimethyl urea, Diuron, Sigma-Aldrich) immediately prior to the 16 h dark adaptation. Light treatments were performed as described above.

### RNA isolation and RT PCR analysis

Total RNA from rosette leaves was isolated with the Spectrum Plant Total RNA Kit (Sigma-Aldrich) with an on column gDNA digestion (Sigma-Aldrich). RNA concentration was determined using NanoDrop ND-1000. First-strand cDNA synthesis was performed with the RevertAid M-MuLV Reverse Transcriptase Kit (Thermo Scientific) with 1 μg of RNA and oligo (dT)_18_ primers. Real-time PCR conditions were: 10 min at 95 °C and 40 cycles of 15 s at 95 °C, 15 s at 56 °C for GLRs or 51 °C for reference genes, and 20 s at 72 °C. The specificity of the PCR products was verified on a dissociation curve. All reactions were run in triplicate. Sequences of primers are given in Additional file 1. Transcript levels of target genes were normalized using the reference genes and factors calculated with geNorm v 3.4 [34].

To choose genes analyzed as part of the irradiation experiments we attempted to detect the expression of all 20 *AtGLR*s in leaves. Isolation of RNA from unilluminated leaves and roots was performed according to the above-described protocol. In eight cases (*AtGLR1.3*, *AtGLR2.1*, *AtGLR2.2*, *AtGLR2.3*, *AtGLR2.4*, *AtGLR2.5*, *AtGLR2.6,* and *AtGLR2.9*) the PCR products were either absent from leaves or two bands were observed while single products of expected length were detected in roots. The list of primers used is given in Additional file 1. Twelve genes which show unequivocal expression in leaves were chosen for further studies.

## Supplementary Information

**Additional file 1.** Sequences of primers used in the study.

**Additional file 2.** Induction factors for mRNA of specific AtGLRs after blue or red light irradiation, showing changes in expression level after irradiations.

## Data Availability

The data are available from Jagiellonian University Repository (https://ruj.uj.edu.pl/xmlui/?locale-attribute=en). The seeds of homozygous T-DNA Arabidopsis mutants are available from the corresponding author.

## Abbreviations

BL: Blue light
FR: Far red light
RL: Red light
WT: Wild type
