# Supplementary material for: Upregulation of GLRs expression by light in Arabidopsis leaves
Source: BMC Plant Biol. 2022 Apr 15;22:197. doi: 10.1186/s12870-022-03535-7 (PMC9013116; doi:10.1186/s12870-022-03535-7)
Supplement: Supplementary file 1 — Additional file 1. Sequences of primers used in the study. [file 12870_2022_3535_MOESM1_ESM.docx]

**Additional file 1A.** Sequences of primers used in the study. Gene-specific primers were designed using NCBI Primer Design. Primers for reference genes: PDF2, SAND, and UBC are based on Czechowski et al. [35]. F denotes a forward primer and R a reverse primer.

| Primer | Sequence (5'–3') | Primer | Sequence (5'–3') |
| --- | --- | --- | --- |
| GLR1.1_F | TGGCTTTGGCTTTATGTTCCAGA | GLR3.4_F | CGAACACCGCTTCAACCAAG |
| GLR1.1_R | TCTCCATGTCTTTCAACATTCCCA | GLR3.4_R | ACTCACCGTGTTCTCCCTATGA |
| GLR1.2_F | GCTCATCGGGAGAAGCTACAAC | GLR3.5_F | TGGCGGCCATTGAAGACATT |
| GLR1.2_R | CGTATTTGTTGCACCGTCATCAT | GLR3.5_R | TCCATTAGCTGCAAAGCTCCC |
| GLR1.4_F | TTGTCTTTGCCCATCGGGAG | GLR3.6_F | GATTAGAAGTGGGTTGGGGGA |
| GLR1.4_R | ACCCGCGTATCTGTTGAACC | GLR3.6_R | GAGGCAATGGTGGAGGAAGT |
| GLR2.7_F | TGACCCCAACGGGTTACTGT | GLR3.7_F | TGCCATTGTCGACGAACTCC |
| GLR2.7_R | CGTCATAGGCCCCGGTGTA | GLR3.7_R | TTAAACGCAAATCCCCAACCAC |
| GLR2.8_F | CGATAACGGGACTTTGGATGC | SAND_F | AACTCTATGCAGCATTTGATCCACT |
| GLR2.8_R | ACCGGCACCATCATAGACAC | SAND_R | TGATTGCATATCTTTATCGCCATC |
| GLR3.1_F | GGGATTTGCATTCCCAAGAGA | PDF2_F | TAACGTGGCCAAAATGATGC |
| GLR3.1_R | GATTTCGAAAGCCATCGGTC | PDF2_R | GTTCTCCACAACCGCTTGGT |
| GLR3.2_F | TCATGTGCCTGACGAATCTGC | UBC_F | CTGCGACTCAGGGAATCTTCTAA |
| GLR3.2_R | GCTTTGGCTTAATCAGTTCCTCAAA | UBC_R | TTGTGCCATTGAATTGAACCC |
| GLR3.3_F | TCGCGCATAGAGAGAACACAG |  |  |
| GLR3.3_R | AAGAGAGCTGCTGAACCGTC |  |  |

**Additional file 1B.** Sequences of primers used in preliminary studies. With these primers, the products of the expected size were obtained in roots while in leaves, the products were either absent or two bands were observed. F denotes a forward primer and R a reverse primer.­­

| Primer | Sequence (5'–3') | Primer | Sequence (5'–3') |
| --- | --- | --- | --- |
| GLR 1.3_F2 | TGCCATACCTCAAAGTTGTCCT | GLR2.4_F1 | TGCATCGACTTCTTTGAGGCT |
| GLR 1.3_R2 | GCCCTTTTGAAACATAAAGCCGA | GLR2.4_R1 | TGCCAATATCGTCGTATCGTT |
| GLR 1.3_F7 | TTCGCGCTTCGATTAGTCCT | GLR2.5_F | AGGAGGCCATCAGAGAGCTT |
| GLR 1.3_R7 | CGCATCATATTTGTCTTTCTGGCT | GLR2.5_R | AGCCAAAGCCATCAGCCTTA |
| GLR 2.1_F2 | TTTGCGCCGAGAGAACGAG | GLR2.5_F5 | TGATGCTGCGTTCGATGAGG |
| GLR 2.1_R2 | TGTTGTAAGAAGCGACGCCA | GLR2.5_R5 | GTGGAAATGCAAAGCCAAAGC |
| GLR 2.1_F7 | GGTAAATCCAGACTTTGATGGGC | GLR2.6_F1 | CCGTCAAGGATGAGAGGGAA |
| GLR 2.1_R7 | TAAGCACTCGTTCTCTCGGC | GLR2.6_R1 | TGCTGTGTAGCTCATGATCCC |
| GLR 2.2_F2 | TGCTCCAAGAGAAAGAGTGC | GLR2.6_F1_5 | GTACCCGTCAAGGATGAGAGG |
| GLR 2.2_R2 | GATGTCAAAAGCGACGCCAA | GLR2.6_R1_5 | TGCTGTGTAGCTCATGATCCC |
| GLR 2.2_F10 | GTCAATTCGGACTTCCGTGG | GLR2.7_F | AGTATACACCGGGGCCTATGA |
| GLR 2.2_R10 | TAAGCACTCTTTCTCTTGGAGCA | GLR2.7_R | CACTCCAGACTCTGTGTACGG |
| GLR2.3_F7 | TGGATTCGGCTTTGTTTTTCCA | GLR2.8_F | CGATAACGGGACTTTGGATGC |
| GLR2.3_R7 | CTCGAGCTCCATTGCCTTTG | GLR2.8_R | ACCGGCACCATCATAGACAC |
| GLR2.3_F1 | CCTCGGGAGATATGATGCGG | GLR2.9_F6 | TGGCTTTGCATTCCCGAAGA |
| GLR2.3_R1 | ACGATCAATCCGACACCTGA | GLR2.9_R6 | TTCGGGAACCATCTGTCCTC |
| GLR2.4_F4 | TGGAAAAACTAACGATACGACGA | GLR2.9_F7 | ATGGTTTTTGCCCACCGTGA |
| GLR2.4_R4 | TAGAGGGACAACCATCCCCA | GLR2.9_R7 | CGCTGTGTAGCTCTGAGTGA |
