# Supplementary material for: Upregulation of GLRs expression by light in Arabidopsis leaves
Source: BMC Plant Biol. 2022 Apr 15;22:197. doi: 10.1186/s12870-022-03535-7 (PMC9013116; doi:10.1186/s12870-022-03535-7)
Supplement: Supplementary file 2 — Additional file 2. Induction factors for mRNA of specific AtGLRs after blue or red light irradiation, showing changes in expression level after irradiations. [file 12870_2022_3535_MOESM2_ESM.docx]

Additional file 2. Induction factors for mRNA of specific *AtGLR*s after blue or red light irradiation. The factors were calculated by dividing relative gene expression levels after red light or blue irradiation by those seen in the dark. Values are provided for wild type plants and for the photoreceptor mutants studied.

| Genotype | Light treatment | *AtGLR1.1* | *AtGLR2.7* | *AtGLR3.1* | *AtGLR3.2* | *AtGLR3.3* | *AtGLR3.5* | *AtGLR3.7* | Columbia background |
| --- | --- | --- | --- | --- | --- | --- | --- | --- | --- |
| WT Col | Red | 4.7 | 6.5 | 6.3 | 6.0 | 2.6 | 3.9 | 8.7 |  |
|  | Blue | 2.5 | 5.2 | 4.5 | 11.2 | 2.8 | 5.9 | 10.2 |  |
|  | | | | | | | | |  |
| *phyA* | Red | 1.5 | 3.8 | 3.7 | 5.4 | 2.1 | 2.1 | 3.9 |  |
|  | Blue | 1.3 | 2.5 | 2.7 | 9.0 | 2.7 | 2.8 | 7.2 |  |
|  | | | | | | | | |  |
| *phyB* | Red | 1.3 | 2.6 | 1.8 | 4.0 | 1.5 | 3.7 | 2.3 |  |
|  | Blue | 1.7 | 6.0 | 2.1 | 12.1 | 5.0 | 10.3 | 5.7 |  |
|  | | | | | | | | |  |
| *phot1/phot2* | Red | 2,6 | 10,3 | 4,5 | 12,0 | 2,6 | 4,8 | 7,4 |  |
|  | Blue | 3,0 | 7,6 | 3,3 | 20,3 | 3,7 | 5,5 | 8,8 |  |
|  | | | | | | | | | Landsberg erecta background |
| WT Ler | Red | 1.8 | 20.3 | 9.3 | 7.9 | 2.5 | 6.5 | 3.6 |  |
|  | Blue | 1.6 | 12.3 | 7.3 | 12.2 | 4.0 | 10.8 | 5.0 |  |
|  | | | | | | | | |  |
| *cry1* | Red | 1.9 | 13.3 | 7.4 | 6.2 | 1.8 | 3.9 | 6.3 |  |
|  | Blue | 2.7 | 16.9 | 6.4 | 6.5 | 2.7 | 5.2 | 5.9 |  |
|  | | | | | | | | |  |
| *cry2* | Red | 2.9 | 5.5 | 7.4 | 4.0 | 1.5 | 3.4 | 3.9 |  |
|  | Blue | 2.8 | 8.3 | 4.9 | 12.1 | 3.2 | 8.0 | 5.3 |  |
|  | | | | | | | | |  |
| *cry1/cry2* | Red | 2.4 | 9.6 | 9.9 | 8.5 | 2.4 | 4.9 | 4.0 |  |
|  | Blue | 3.9 | 9.4 | 8.0 | 10.0 | 2.3 | 4.9 | 3.6 |  |
